# One organ to infect them all: the *Cuscuta* haustorium

**DOI:** 10.1093/aob/mcae208

**Published:** 2024-12-02

**Authors:** Vasili A Balios, Karsten Fischer, Thomas Bawin, Kirsten Krause

**Affiliations:** Department of Arctic and Marine Biology, UiT The Arctic University of Norway, Tromsø, Norway; Department of Arctic and Marine Biology, UiT The Arctic University of Norway, Tromsø, Norway; Department of Arctic and Marine Biology, UiT The Arctic University of Norway, Tromsø, Norway; Department of Arctic and Marine Biology, UiT The Arctic University of Norway, Tromsø, Norway

**Keywords:** Dodder, haustoriogenesis, haustorium evo-devo, interspecies connections, natural graft, parasitic plant

## Abstract

**Background:**

Research on the parasitic plant genus *Cuscuta* has flourished since the genomes of several of its species were published. Most of the research revolves around the iconic infection organ that secures the parasite’s sustenance: the haustorium. Interest in understanding the structure–function–regulation relationship of the haustorium is based as much on the wish to find ways to keep the parasite under control as on the opportunities it offers to shed light on various open questions in plant biology.

**Scope:**

This review will briefly introduce parasitism among plants, using the genus *Cuscuta* as the main example, before presenting its haustorium alongside the terminology that is used to describe its architecture. Possible evolutionary origins of this parasitic organ are presented. The haustorium is then followed from its initiation to maturity with regard to the molecular landscape that accompanies the morphological changes and in light of the challenges it must overcome before gaining access to the vascular cells of its hosts. The fact that *Cuscuta* has an unusually broad host range stresses how efficient its infection strategy is. Therefore, particular consideration will be given in the final section to a comparison with the process of grafting, being the only other type of tissue connection that involves interspecific vascular continuity.

**Conclusions:**

Studies on *Cuscuta* haustoriogenesis have revealed many molecular details that explain its success. They have also unearthed some mysteries that wait to be solved. With a better understanding of the complexity of the infection with its combination of universal as well as host-specific elements that allow *Cuscuta* to parasitize on a wide range of host plant species, we may be many steps closer to not only containing the parasite better but also exploiting its tricks where they can serve us in the quest of producing more and better food and fodder.

## INTRODUCTION

Within the plant kingdom there is a sizeable group of plants that have adopted the lifestyle of a parasite by feeding on other plants. An estimated 1 % of all land plant species are believed to live a parasitic lifestyle (Heide-Jørgensen, 2008) and this group is anything but homogeneous: its members come from varying phylogenetic backgrounds, have different trophic strategies (i.e. hemi- or holoparasitic), have developed different morphological reductions depending on the point of infection (above or below ground), and vary in the amount of intimacy they share with their host (i.e. the amount of the parasite that is endophytic). Nevertheless, all of them have one common signature feature: the haustorium as the organ responsible for the infection process (Kuijt, 1969). The haustorium is a unique organ that is not found in non-parasitic plant species. Although the name suggests a relationship with the better known fungal haustoria, there is little that the multicellular haustorium of parasitic plants has in common with them except for their function in nutrient acquisition (Mayer, 2006).

Parasitization can have dire consequences on crop yield and quality, which is why considerable research efforts have been, and still are, devoted to pinpoint how the parasite’s distribution can be confined, and infection impact mitigated. Haustorium initiation and maturation represent critical stages in the lifecycle of a parasitic plant, and their understanding is pivotal to the ability to control the parasite. Our current perception of haustoriogenesis stems from the investigation of about half a dozen parasitic plant species from the families Orobanchaceae (all root parasites) and Convolvulaceae (shoot parasites) (reviewed for instance by Yoshida *et al.*, 2016; Kokla and Melnyk, 2018; Furuta *et al.*, 2021; Jhu and Sinha, 2022; Hartenstein *et al.*, 2023; Kirschner *et al.*, 2023). From these systems we can deduce that complex molecular and cellular processes orchestrate the establishment of the intimate haustorial connections, which by some parasitic species are established with a vast variety of host plants. Recently, two independent studies by Vogel *et al.* (2018) and Sun *et al.* (2018) have provided valuable insights into the genomes and the molecular adaptations associated with parasitism in *Cuscuta* species. These studies caused a wave of molecular studies that highlighted the complex interplay between gene family contraction and expansion, positive and relaxed purifying selection, as well as horizontal gene transfer. All these factors apparently created a molecular environment in which re-wiring of molecular pathways that resulted in parasitic structures and functions (such as the haustorium) would have been promoted. Most recently, two more genomes (for *Cuscuta epithymym* and *C. europaea*) have been published (Neumann *et al.*, 2023).

**Fig. 1.**
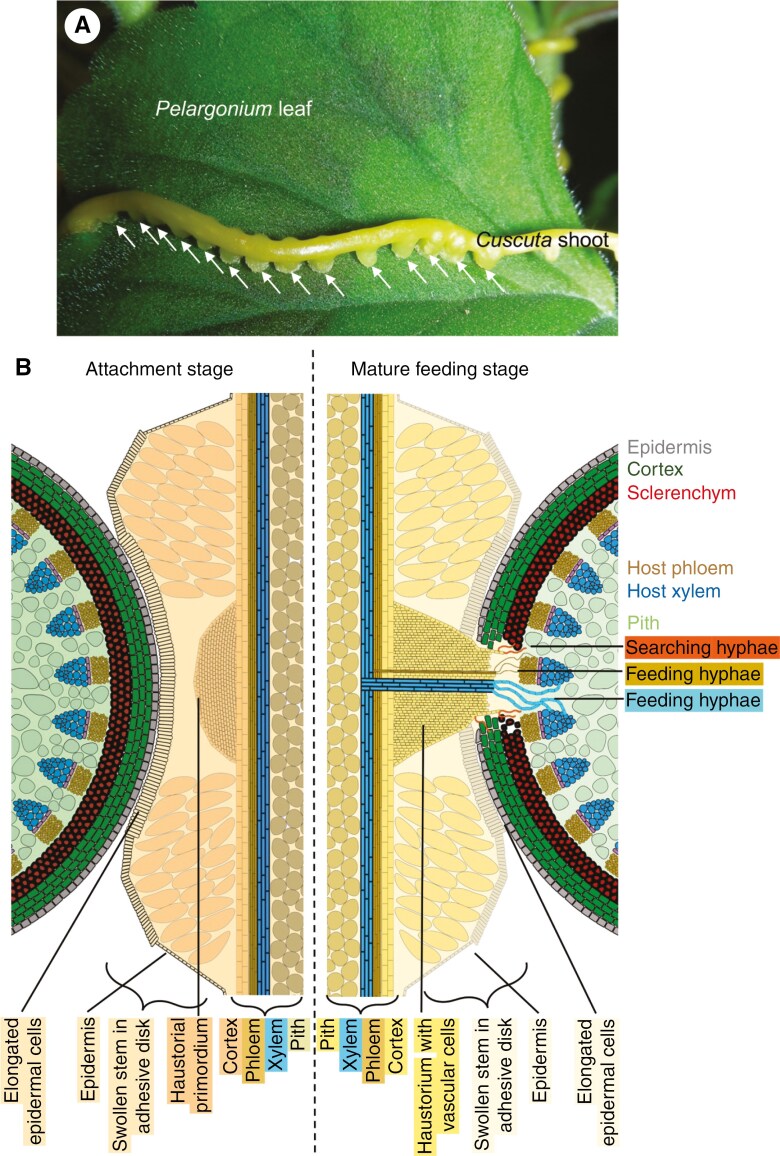
The haustoria of *Cuscuta*. (A) Photo of a *Cuscuta* species on a *Pelargonium* leaf with multiple haustoria lined up one after another (marked by white arrows). The haustoria’s adhesive disc as an exophytic telltale sign for an infection is clearly visible on the *Cuscuta* stem as a unilateral swelling. (B) Schematic views of early (left) and late (right) haustorial developmental stages showing *Cuscuta* (orange/yellow base colour) in longitudinal section, on a host stem (green base colour) depicted in cross-section. Only cells with shapes deviating from the common cortical parenchymatic cells are schematically represented.

While this recent wave of molecular research on *Cuscuta* has yielded invaluable insight into a complex organ and its function, it must be acknowledged that the interpretation of the many sets of data is facilitated by a solid basis of knowledge on *Cuscuta* haustorium architecture dating back many decades (Hartenstein *et al.*, 2023). Initially, the curiosity in this genus may have been sparked by the highly conspicuous and unusual looking haustoria whose clustered appearance in the host foliage and stems is difficult to overlook (Fig. 1A). The continued fascination, however, seems to stem from several intriguing features displayed by this parasite that have become obvious during a number of older seminal studies. First and foremost is the ability of the parasite to develop a cytoplasmic continuity and vascular connections with a diverse range of hosts from a large variety of taxa (Vogel *et al.*, 2018). That these include herbs, bushes as well as trees is a capacity that could be exploited for agricultural purposes if it were better understood. Equally fascinating is the question of why most host plants do not seem to be able to identify the parasitic cells of the haustorium as intruding foreign tissue, sparking investigations into cell identity, cell-wall protection and cell-wall degradation.

Information on the haustoriogenesis from parasitic lineages beyond the Orobanchaceae and Convolvulaceae is much more scarce (e.g. Pielach *et al.*, 2014; Teixeira-Costa, 2021), so that we are not yet in a position to draw overarching conclusions on the iconic organ of parasitic plants. This review will therefore focus primarily on the haustorium of *Cuscuta*, exploring its potential evolutionary origin and covering its development from initiation to maturity with the important vascular connections. It provides an overview of the molecular landscape, with a particular emphasis on cell-wall dynamics and solute transport processes. It is meant to complement some recent reviews that have delved deeper into other areas, such as the morphological or molecular comparisons with other parasitic groups, the interactions with hosts, and the host resistance mechanisms that exist against parasites (Jhu and Sinha, 2022; Albanova *et al.*, 2023; Ashapkin *et al.*, 2023; Hartenstein *et al.*, 2023; Kirschner *et al.*, 2023; Bawin and Krause, 2024), presenting an emerging view of the haustorium as being a natural graft and, thus, a valuable resource for crop improvement by grafting.

## HAUSTORIAL TERMINOLOGY

The term ‘haustorium’ was coined at the beginning of the 19th century to describe suction structures in spermatophytes that live of other plants. Acknowledging that this nutrient- and water-absorptive organ can be morphologically quite diverse, it was essentially used wherever a tissue bridge physically and physiologically connects a parasite and its host (Kuijt, 1969). In older literature, the term ‘haustorium’ was used only in reference to the endophytic tissue, while the exophytic structures that are responsible for the attachment of the parasite to the host surface were referred to as ‘upper haustoria’ or ‘prehaustoria’. In some articles the endophytic part of the haustorium is also referred to as ‘haustorium proper’ or ‘lower haustorium’. In the last two to three decades, a less discriminative use of the word haustorium has become increasingly popular where the entire infection organ including the adhesive and suction structures are referred to as the haustorium. In this review we will use the more precise distinction of prehaustorium and haustorium proper only where it is appropriate to distinguish the two haustorial parts, and otherwise use the general term ‘haustorium’ for the entire composite infection structure encompassing all endophytic and exophytic tissue of the parasite up to the interface with the host (but excluding host tissue).

Each *Cuscuta* haustorium develops from a tertiary meristem that is formed in the inner cortical layers of the *Cuscuta* stem on the host-facing side and around 2–10 cm below the growing apical tip. This initially disc-like collection of dividing cells is referred to as the ‘haustorial primordium’ (Lee, 2007). Its placement relative to the vascular tissue may vary to some extent depending on the *Cuscuta* stem diameter, pointing tentatively to a dependence on exogenous rather than endogenous signals. This is supported by the fact that the initiation of a haustorial primordium is tightly correlated with a deformation of the flat brick-shaped epidermal cells of *Cuscuta* into club-shaped, vertically elongated secretory cells at the point of host contact (Fig. 1B). By periclinal and anticlinal cell divisions in the inner cortex layers, the haustorial primordium grows rapidly, generating a cone of cells that eventually emerges from the stem of *Cuscuta* (Svubova *et al.*, 2017; Kirschner *et al.*, 2023). While this happens, cortical cells around the expanding meristem elongate in the direction of the host, creating a swollen ring of tissue that becomes clearly visible to the naked eye (Fig. 1A). This ring or disc is referred to as ‘adhesive disc’, ‘appressorium’ or ‘holdfast’. As these names indicate, it anchors the parasite to the host surface with a thin layer of glue. The developing haustorium proper, emerging from the middle of the adhesive disc, can proceed its growth directly into the adhering tissue of the host. In the host, long and flexible tentacle-like cells termed ‘hyphae’ start to grow outward from its tip and sides into the host tissue (Fig. 1B). These hyphae, which are the cells that are ultimately responsible for nutrient and water absorption, turn from so-called ‘searching hyphae’ into ‘feeding hyphae’ upon establishing connections with host vascular cells (reviewed by Hartenstein *et al.*, 2023).

## THE EVOLUTIONARY ORIGIN OF THE HAUSTORIUM

Lyko and Wicke (2021) suggested three major phases in the evolution of parasitic plants. Phase I involves functional innovations that enable a plant to exploit another plant as a host. Phase II is characterized by parasitic specialization, during which molecular–evolutionary constraints on dispensable, host-complemented molecular processes are relaxed. Meanwhile, in phase III interactions between the parasitic plant and its host are tightened and optimized for efficiency. The idea that the development of a haustorium could have been the prerequisite for other morphological adaptations in the evolution of parasitic plants was voiced earlier by Furuhashi *et al.* (2011). They suggested that the evolution of haustoria in *Cuscuta* occurred in parallel with other changes in its anatomy. According to the authors, due to mechanical stress, a strong contact signal by twining around a host might have led to reduced chlorophyll levels and failure to develop proper roots and leaves. During this stage of evolution, individuals that developed tissues capable of nutrient extraction would have had a competitive advantage.

The evolutionary origins of parasitic plant haustoria have been the subject of intense debate over decades. A current consensus is that haustoria in the different lineages of parasitic plants independently evolved as specialized tissues and cannot be understood as a modified, strict homologue of another plant organ because clear parallels do not exist (Kuijt, 1969; Furuhashi *et al.*, 2011; Teixeira-Costa, 2021). The shared features in haustorium development among parasitic lineages (adhesion, intrusion, connection) could be due to similar patterns of expression of homologous regulatory genes (Teixeira-Costa, 2021). Although haustoria perform the same basic functions as a root system (i.e. providing mechanical anchorage and facilitating the uptake of water and mineral nutrients), the relationship between haustoria and roots remains obscure when taking the developmental and anatomical levels into consideration. Lateral and adventitious roots in angiosperms develop from meristematic cells connected to the vascular system (referred to as an endogenous origin), which differs from the haustorium induction in *Cuscuta* that happens in the inner cortical layers, unconnected to the parasite’s vasculature (Svubova *et al.*, 2017; Kirschner *et al.*, 2023). Since the epidermal cells are somehow connected with the initiation of haustoriogenesis, possibly by relaying information about the presence of a host, some authors have referred to the haustorial origin in an ontogenetic context as exogenous to contrast it with the endogenous origin of roots (Shimizu and Aoki, 2019; Teixeira-Costa, 2021). The internal anatomy of parasitic plant haustoria is, furthermore, not organized into sectors that can be classified as either root-like or stem-like (Teixeira-Costa, 2021). Adding to the uncertainty, molecular analyses in *Cuscuta* revealed the involvement of genes in haustorium development that control root (Sun *et al.*, 2018) as well as shoot (Alakonya *et al.*, 2012) formation in non-parasitic plants. Vogel *et al.* (2018) reported that many genes related to root functions were lost in *C. campestris* which would be a logical consequence of its lifestyle that is detached from the soil and, therefore, root-independent. However, not all root-related genes were lost, and it appears that at least some of the genes that were retained have been repurposed for haustorium development.

Expanded families of genes that have more family members than in related autotrophic species, among them those with functions in auxin signalling that plays a role in root and shoot development, were identified in *C. australis* (Sun *et al.*, 2018) and in *C. campestris* (Vogel *et al.*, 2018), entailing the potential for functional diversification via sub- or neo-functionalization. In addition, *Cuscuta* parasites were suggested to compensate for genomic losses by incorporating and expressing genetic material from their hosts in a process known as horizontal gene transfer (HGT) (Vogel *et al.*, 2018; Yang *et al.*, 2019). Experimental evidence for the functional relevance of transferred genes in the infection process is, however, still lacking, and thus HGT does not offer any clear answer to the question of which organ dodder haustoria have originated from.

Intriguing parallels to the haustorial organ can be found in the adhesive structures that secure climbing plants to vertical surfaces. Some of these have their origin in adventitious roots, as for example in ivy (*Hedera helix*) or in the climbing fig (*Ficus pumila*) (Groot *et al.*, 2003; Melzer *et al.*, 2010). A conspicuous similarity between the *Cuscuta* haustorium, specifically the adhesive disc that forms prior to host penetration, and the climbing roots of ivy is the secretion of a sticky mucilage. In ivy this mucilage was found to be composed of pectic rhamnogalacturonan-I (RG-I) polysaccharide domains and nanospherically arranged arabinogalactan proteins (AGPs) (Huang *et al.*, 2016). De-esterified pectins, but not AGPs, were also reported to be secreted from the epidermal cells of the adhesive discs of *Cuscuta* haustoria, where they, too, form the thin cement layer between the parasite and the host surface (Vaughn, 2002).

Other climbers such as the Virginia creeper (*Parthenocissus quinquefolia*) or the climbing passion flower (*Passiflora discophora*) rely on short shoot-derived tendrils containing adhesive discs or pads at the end for climbing (Bowling and Vaughn, 2008; Klimm *et al.*, 2022). Immunocytochemical analyses of their adhesive compounds revealed again linear RG-I as the main constituent and, in addition, callose that may be fortified with lignin in older tendrils of *Parthenocissus quinquefolia* (Bowling and Vaughn, 2008), indicating that the similarities in the chemical composition of the adhesive is probably due to the chemical properties of pectin and the general ability by all cells to produce and secrete this abundant cell-wall constituent. Although the Convolvulaceae as a family do not possess tendrils, it may be worthwhile to compare the molecular regulation in these climbing structures with that in *Cuscuta* haustoria in order to identify the extent of convergence in plant adhesive structures, particularly because they share another intriguing feature: their sensitivity to touch (Meloche *et al.*, 2007; Sousa-Baena *et al.*, 2018) (see section below). In summary, whether the haustorium of *Cuscuta* can be interpreted as a structure of its own kind (Furuhashi *et al.*, 2011) or a mosaic of different structures (Teixeira-Costa, 2021) or yet something else still awaits clarification.

## THE REINS OVER HAUSTORIUM INDUCTION ARE HELD BY ABIOTIC CUES

It is vital for a parasitic plant to locate the presence of a suitable host and to induce a haustorium in locations where they can infect the host. To achieve this, there are a number of different cues that the parasite has been reported to react to. These can be grouped into chemotropic, thigmotropic and phototropic signals.

### Chemotropism

It is well established that hosts give away their presence to root parasitic plants by releasing chemoattractants into the soil. These so-called haustorium-inducing factors (HIFs) are a diverse group of compounds that consist mostly of phenolic substances and cytokinins (Goyet *et al.*, 2019). They are detected by the parasite’s roots, which respond to the HIF gradient by directional growth and haustorium induction. As a shoot parasite that is not in contact with the soil, *Cuscuta* is not exposed to the classical HIFs. The contribution of host-derived chemicals to the induction or formation of a haustorium is in general much less well characterized for shoot parasites. Nevertheless, a selectivity in favour of more rewarding hosts with higher nutritional quality was reported multiple times for *Cuscuta*. Although this appears to affect its foraging behaviour (summarized by Bawin and Krause, 2024), it is to date not really clear what attracts the parasite. One possibility are plant volatiles. Volatile substances are released by many plants, and they have indeed been implicated in one study in guiding growth of *Cuscuta pentagona* seedlings towards compatible host plants (Runyon *et al.*, 2006). While that study showed that *Cuscuta* is able to perceive these substances and react by shoot elongation towards them in a controlled laboratory experiment, it is difficult to conceive how the comparatively slow perception (in the range of days for growth responses by *Cuscuta*) would pan out in a natural setting where many individual plants, each releasing its own volatile bouquet, are present and where wind or other abiotic factors would erase the directional information faster than *Cuscuta* can grow towards it or infect. Moreover, the global transcriptional landscape during haustorial initiation appears similar whether a live host is present or not (Bawin *et al.*, 2022), revealing to date no evidence for a perceptive mechanism for chemoattractants. This changes first when *Cuscuta* commits to haustorial penetration of the host, where clear-cut differences in gene expression can be related not only to host presence but also to their cell-wall composition (Bawin *et al.*, 2023). This indicates that chemoattractants play at best a very minor role in directing *Cuscuta* growth towards a host inducing haustoriogenesis.

### Thigmotropism

**Fig. 2.**
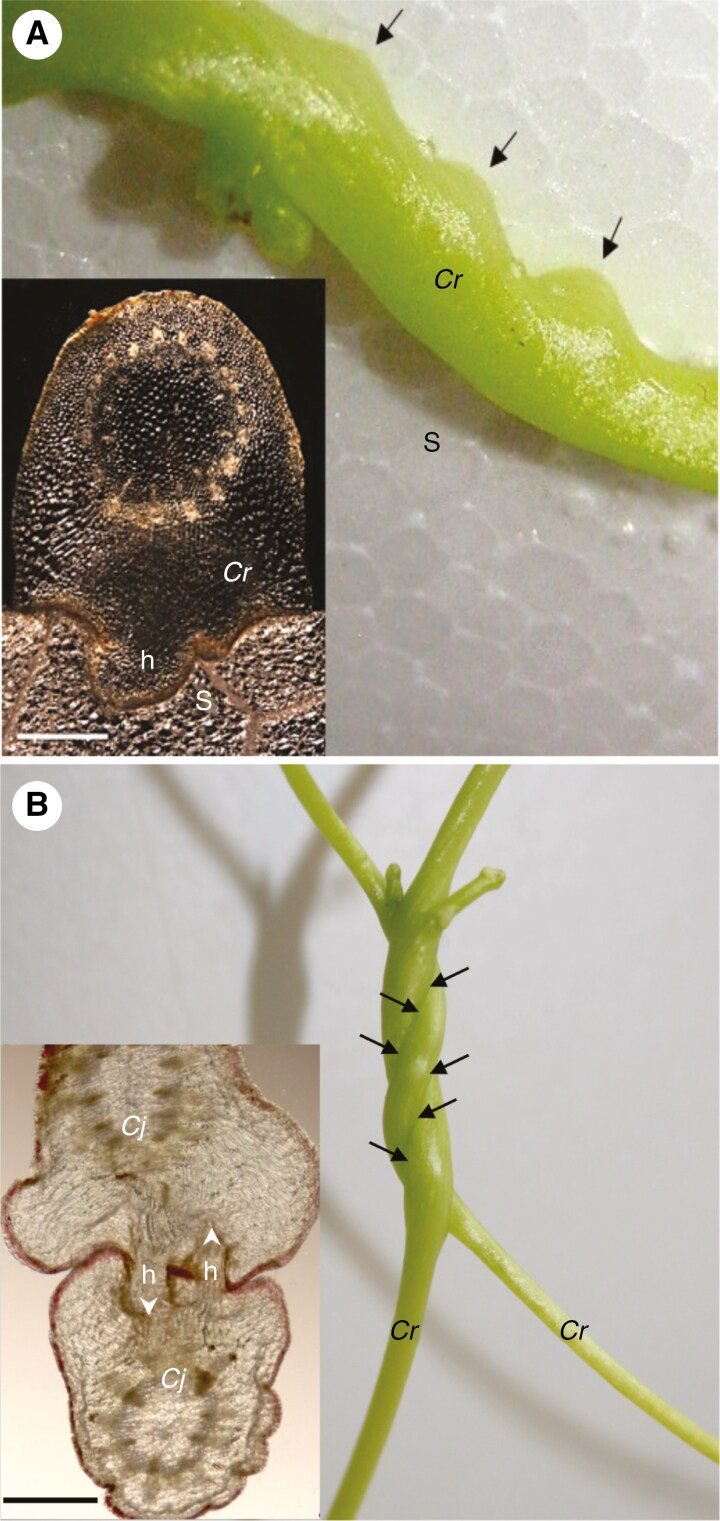
Non-host specificity of haustorium formation by *Cuscuta*. (A) *Cuscuta* species on Styrofoam. A cross-section of the ‘infection site’ with the invading haustorium is depicted in the inset. (B) Self-infecting *Cuscuta* species. A cross-section of a bilateral infection site with two haustoria each invading the opposite stem is seen in the inset, where arrowheads indicate direction of haustorial growth. *Cr* = *C. reflexa*; *Cj* = *C. japonica*; h = haustorium; S = Styrofoam. Arrows point to the haustoria in each image. Scale bars in both insets are 1000 µm.

Many of the agriculturally most damaging *Cuscuta* species, among them the sequenced species *C. campestris*, *C. australis*, *C. epithymym* and *C. europaea*, thrive on a large variety of hosts, which suggests that they would fare best by using very general signposts for the presence of plants, rather than host-specific chemical signatures. Moreover, *Cuscuta*’s readiness to commit to haustorium development on metal rods, wooden sticks or other non-plant surfaces (Fig. 2A), as well as its cannibalistic tendency to self-infection (Hong *et al.*, 2011; Kaiser *et al.*, 2015) (Fig. 2B) are evidence for its pronounced promiscuity, and contradict the notion of a strong guidance by chemical attractants, but rather react more to a touch stimulus. Indeed, thigmotropic cues were found to strongly influence host-finding and haustoriogenesis (Ihl and Wiese, 2000), and may suffice to trigger the latter without the need for other stimuli. It should be noted that touch-sensitivity was restricted to the regions just below the shoot apex which is also the area where the most intensive shoot elongation and haustoriogenis take place (Ihl and Wiese, 2000; Olsen *et al.*, 2016*b*). While as yet there are little data outlining the signalling cascades in response to touch in *Cuscuta*, Jhu and Sinha (2022) have proposed a model with potential players that is based on current knowledge from non-parasitic model plants and that may serve as a guideline for future studies. It postulates the possible involvement of Ca^2+^ signalling and membrane potential changes upstream of the activation of transcription factors. Based on knowledge about touch-induced responses from non-parasitic plants (Fernandez-Milmanda, 2024), it can also be hypothesized that the phytohormones jasmonic acid and ethylene as well as gibberellins are involved at an early stage in the induction process.

### Phototropism

In contrast to the still enigmatic stimulation of *Cuscuta* haustoriogenesis by touch, the fact that light in the far-red (FR) spectral range induces nutation of the *Cuscuta* shoots, tight coiling around the host stem and the formation of haustoria, in addition to inducing positive phototropism of germinating *Cuscuta* seedlings and shoots, has received considerably more attention (e.g. Furuhashi *et al.*, (2021)). Rates of host location and attachment by *Cuscuta* was shown to be reduced under light conditions with a high red:FR ratio (Johnson *et al.*, 2016). Light conditions under dense green foliage have a lower red-light proportion in relation to FR light as it is absorbed by chlorophyll, so the red:FR ratio, in fact, provides the parasite with an efficient means to detect areas with high host abundance, or in other words, denser vegetation.

Plants are known to possess a complex array of light receptors, including phytochromes (PHYA-E) for red and FR light, as well as cryptochromes (CRY1-2) and phototropins (PHOT1-2) as sensors of blue light (Jing and Lin, 2020). These receptors mediate light perception and activation of downstream signalling events that constantly regulate the plant transcriptome with additive, synergistic or antagonistic effects, leading to differential growth responses between tissues and organs via transcription factors such as the master regulator ELONGATED HYPOCOTYL 5 (HY5) and a number of PHYTOCHROME INTERACTING FACTORs (PIFs) (Jing and Lin, 2020; Cheng *et al.*, 2021; Huber *et al.*, 2021). The involvement of phytochromes in *Cuscuta* haustoriogenesis was postulated early on (Furuhashi *et al.*, 1997) but the unconventional effect of the red:FR ratio in *Cuscuta*, which seeks rather than avoids shade, suggests that critical evolutionary advancements occurred downstream of the initial signals and receptors. These have not yet been satisfactorily unravelled. The current model of light perception in *Cuscuta* assumes that phytochromes switch towards their inactive (PR) form under FR light, thus relieving their interaction partners from restrictions, which in turn activate the expression of genes that are required to initiate haustorium development (Furuhashi *et al.*, 2011; Jhu and Sinha, 2022).

The phytohormone cytokinin promotes parasitism in a light-dependent way (Rajagopal *et al.*, 1988; Haidar *et al.*, 1998; Furuhashi *et al.*, 2021) and was therefore suggested to act downstream of light perception (summarized by Furuhashi *et al.*, 2021). Cytokinin interacts with low auxin concentrations as a prelude to haustorium formation and may bypass the need for light and mechanical stimuli to initiate haustorium development when exogenously applied (Ramasubramanian *et al.*, 1988; Haidar *et al.*, 1998). How and if light, mechanical signals and phytohormones converge on a common pathway at the molecular level that translates into an induction of the haustorial meristem and down the line into an infection attempt is another unanswered but intriguing question.

## THE ALPHA AND OMEGA OF PARASITISM: GAINING ACCESS TO THE HOST

**Fig. 3.**
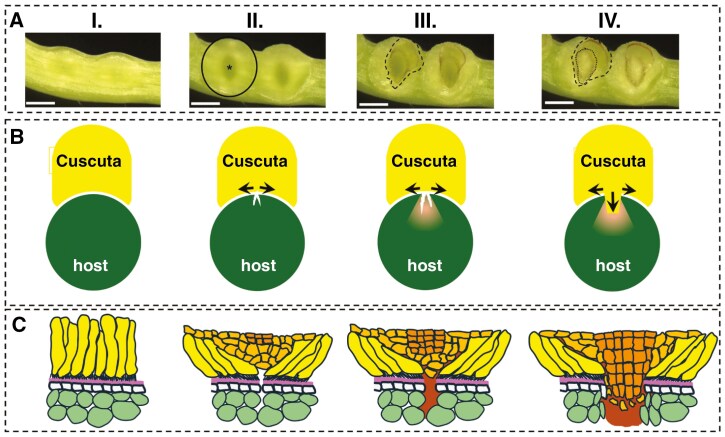
Mechanistic model of host penetration by *Cuscuta*. (A) Micrographs from a time lapse series of two infection sites developing against a translucent Petri dish, starting at the earliest stage where haustoriogenes is irreversible (stage I), and where the unilateral stem swelling as telltale sign is visible. The development of the adhesive disc (stage II), the mucilaginous secretion (stage III) and the tip of the emerging haustorium (stage IV) are outlined (black ring, stippled line and dotted line, respectively). A central gap in the adhesive disc that widens over the course of the attachment and early penetration stage is visible from stage II (asterisk). (B) Schematic depiction of the same stages on a host, seen from a cross-sectional perspective. The widening gap in the adhesive disc from stage II on together with the adhesive forces between the surface of the parasite and the host through secreted pectins is believed to induce micro-fissures by radial tension in the cuticle and epidermis of the host. Secreted degrading enzymes in the mucilaginous drops are able to enter into the space and soften the host tissue (stage III). The emerging haustorium (stage IV) exerts further perpendicular pressure on the host whose cell-wall integrity is already compromised by the discharge of cell-wall-degrading enzymes. (C) Detailed schematic view of the process shown and described in B.

Host stems, petioles and pedicels, which belong to the preferred infected host tissues, are in principle well protected against intruders by epicuticular waxes, the cuticle and the epidermal cell layer, whose cells adhere tightly to each other and form a mechanically strong border. The adhesion strength of cells depends on a variety of factors, such as age and physiological condition of the plant (affecting, for example, turgor pressure) as well as the chemical composition of the cell wall (Cosgrove, 2018). The epidermis evolved to withstand considerable forces, both from within the plant and from the outside. The haustorium was reported to use enzymes to soften this strong barrier (Mayer, 2006) and mechanical force to intrude the host stem. While the proportional contribution of both complementary processes to host penetration has to our knowledge never been systematically assessed, macro- and microscopic observations of developing infection sites in real time, for example by time lapse analysis and in host-free systems (Fig. 3A), have added many details that help to construct a mechanistic model of the different stages of host infection (Fig. 3B, C), which will be described here in more detail.

As already briefly mentioned, a very early change in the development of a haustorium is the transformation of the epidermis on the side of the *Cuscuta* stem proximal to the host into a gland-type surface (Sultana *et al.*, 2021). Club-shaped cells start producing a glue that adheres the parasite firmly to the host surface at the sites where the haustorium proper will emerge (Fig. 3A–C, stage I). This happens before the prehaustorium has reached its final dimensions. While the haustorial meristem is producing haustorial cells by lateral and radial cell divisions, it is concomitantly pushing the adhesive disc apart, leaving a gradually widening gap that is flanked by the adhesive cells. This is assumed to create a very localized lateral tension force on the host surface that is able to pull host epidermal cells apart. In this way, tiny micro-ruptures can be created in the host (Fig. 3A–C, stage II) that serve as entry points for cell-wall-decomposing enzymes that are released by the parasite at the same time. A mucilaginous exudate that is released as a coherent droplet from the central gap preceding the emergence of the haustorium proper (Fig. 3A, stage III) contains carbohydrate active enzymes (CAZymes) as reported in earlier literature (Nagar *et al.*, 1984; Johnsen *et al.*, 2015). These enzymes may enter the host tissue through the micro-fissures and start weakening the cell walls (Fig. 3B, C, stage III). The growing haustorium then exerts perpendicular and radial pressure through its growing body of cells, thereby widening the cracks and pushing apart the host cells whose adhesion strength is already compromised by cell-wall-degrading enzymes. The mucilage layer is likely to aid the emerging haustorium proper with its intrusion by providing lubrication (Fig. 3B, C, stage IV), similar to what has been observed in other types of intrusive cell growth in plants (see, for example, the recent review by Baba and Verger, 2024). A similar mixture of mechanical and enzymatic action on the host tissue, but at a much smaller scale, is exerted by the tentacle-like hyphae that develop when the haustorial body has ceased its growth. At this point the haustorium proper has reached dimensions of up to 0.7 × 1.5 mm in *C. campestris*, or even larger in some of the sturdier *Cuscuta* species such as *C. reflexa* (Johnsen *et al.*, 2015). The combined force from the radial expansion and the perpendicular force of the inward growing haustorium proper is large enough to even sever a protective sclerenchyma, if the host has one.

## THE MOLECULAR KIT FOR HAUSTORIUM INDUCTION AND DEVELOPMENT

**Fig. 4.**
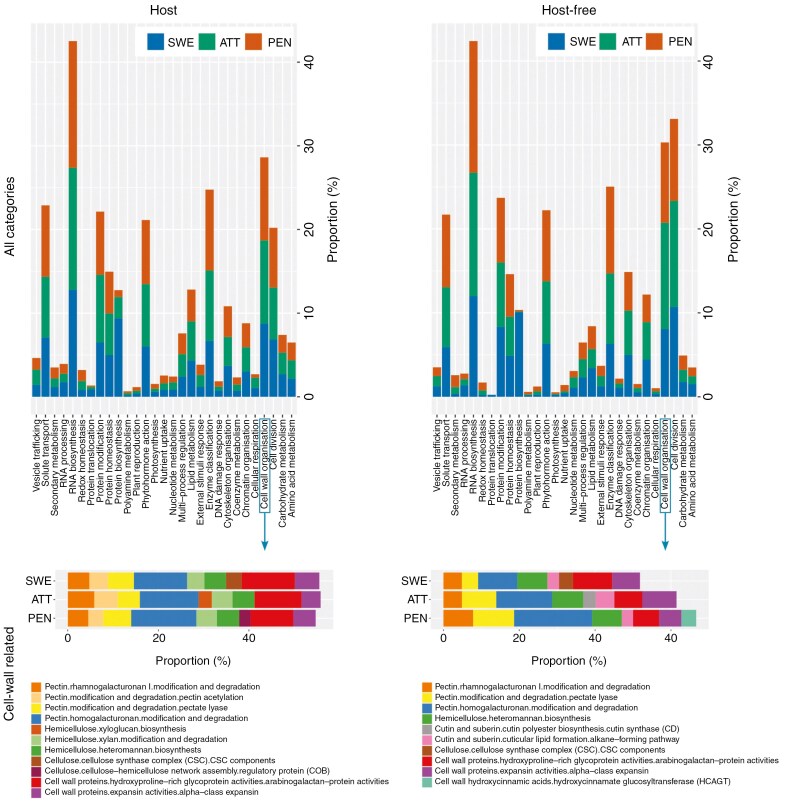
Expression patterns in host-associated haustoria versus host-free haustoria of *Cuscuta*. The bar plots on the top show the proportion of functionally annotated genes (MapMan) that are upregulated in the swelling (SWE), attaching (ATT) and penetrating (PEN) stages relative to a non-infective stem when the parasite is grown on a host (left) or without a host (right). The bar plots at the bottom focus on cell-wall-related genes in the respective systems and stages. After Bawin *et al.* (2023).

Omics studies from the past decade have portrayed a general picture of the molecular pathways that are at play during the different stages of haustoriogenesis in *Cuscuta*, and revealed a diverse array of genes involved in signal perception, transcriptional regulation and morphogenesis, which potentially govern the intricate developmental programme leading to haustorial formation (Ranjan *et al*., 2014; Ikeue *et al*., 2015; Shimizu and Aoki, 2019; Kaga *et al*., 2020; Jhu *et al*., 2021*a*, *b*; Bawin *et al*., 2022, 2023). Much of what is known about the molecular processes driving haustoriogenesis is indeed inferred from transcriptomic studies, and a detailed description of the transcriptional landscape during haustoriogenesis in *Cuscuta* and other parasites is provided in Ashapkin *et al*. (2023). Major changes in expression dynamics were repeatedly observed during the transition from vegetative tissues to a growing infective structure, supporting the involvement of numerous regulatory processes downstream of the light/touch signals (Fig. 4). Specifically, INDOLE-3-ACETIC ACID INDUCIBLES (IAAs), AUXIN RESPONSE FACTORS (ARFs) and SMALL AUXIN UPREGULATED (SAUR)-like genes, involved in auxin response and signalling, are among the genes being regulated during haustorium formation. Besides genes linked to the usual auxin and cytokinin phytohormones, a massive (yet under-investigated) presence of signalling peptides and their receptors from the onset of haustorium development is also indicated in transcriptomic studies (Bawin *et al*., 2023). It is not unlikely that some of them may be deployed by the parasite to counteract the hosts’ immune response. The strong transcriptional upregulation of genes coding for berberine bridge enzyme-like (BBE-like) proteins at the attachment and penetration stages would support this notion. BBEs are known to neutralize oligosaccharides originating from the degradation of (hemi-)cellulose that can act as damage-associated molecular patterns (Scortica *et al.*, 2023). A variety of transcription factors from different families including some that are known in non-parasitic plants to promote the development of lateral organs (Jhu *et al.*, 2021*b*; Bawin *et al.*, 2023) are expressed and control the downstream successions of gene regulation. The best described example in *Cuscuta* so far is CcLBD25, an ASYMMETRIC LEAVES2/LATERAL ORGAN BOUNDARIES (AS2/LOB) transcription factor, that was linked to haustorium initiation, pectin degradation and development of searching hyphae (Jhu *et al.*, 2021*b*). An ethylene-dependent regulatory module was further suggested to modulate the transcription of cell-wall enzymatic genes during haustorial invasion (Yokoyama *et al.*, 2022) but the roles of most transcription factors and other early induced proteins in the context of haustorial development and parasite–host interactions are unfortunately still largely unknown. Post-transcriptional regulation is another important, yet untapped aspect to investigate in future studies. For instance, proteinases are expressed along with cell-wall-degrading enzymes (CWDEs) (Rautengarten *et al.*, 2008), and could play a role in modulating the spatial accumulation and activity of CWDEs at the host–parasite interface in addition to degrading proteinaceous constituents from the host as was demonstrated in other biological contexts. These findings highlight the need for integrative research into the intricate signalling and regulatory mechanisms driving haustoriogenesis, filling the proteomic and metabolic gaps, and revealing how gene expression dynamics translate into functional products and interactions of these.

Cell-wall-related genes play multifaceted and pivotal roles throughout haustoriogenesis. There is a growing body of evidence that enzymes associated with the cell wall are vital in the interactions between *Cuscuta* species and their hosts (Ranjan *et al.*, 2014; Ikeue *et al.*, 2015; Olsen *et al.*, 2016*b*; Jhu *et al.*, 2021*a*; Yokoyama *et al.*, 2022; Bawin *et al.*, 2023; Edema *et al.*, 2024). Genes involved in cell-wall formation and expansion are notably present in early haustoriogenesis in *Cuscuta* (Olsen *et al.*, 2016*b*; Bawin *et al.*, 2022, 2023) (Fig. 4) corroborating earlier cell-wall profiling studies (Johnsen *et al.*, 2015). This pattern is shared with other parasitic groups (Yang *et al.*, 2015; Zhang *et al.*, 2015; Yoshida *et al.*, 2019), and can be interpreted as a common developmental pattern in growing organs where cell division and expansion take place. One protein family that seems to play a central role during haustorial development are the expansins (Ranjan *et al.*, 2014; Bawin *et al.*, 2022) (Fig. 4). These proteins contribute to cell-wall loosening and cell expansion – both processes that are fundamental to the morphological changes in the haustorial primordium. Other genes maintaining high expression levels throughout haustorium development encode, for instance, wall-related fasciclin-like arabinogalactan (FLA) proteins (Olsen *et al.*, 2016*b*; Bawin *et al.*, 2022, 2023). FLA genes are developmentally regulated in the adhesive disc and hyphae (Hozumi *et al.*, 2017), both of which are cell types that are in close contact with the host but nevertheless with different functions. It is not known if the FLA proteins fulfil the same or different functions in these different cell types.

As haustorial development progresses from the early swelling stage to an actively attaching or intruding haustorium, genes from a number of enzymatic families including cell-wall-modifying and cell-wall-degrading genes, amongst others, become involved. Transcripts of numerous genes encoding cell-wall-degrading enzymes, such as endoglucanases, pectate lyases, xylanases and mannanases, were found to be highly abundant in the final infection stages of *Cuscuta* (Ranjan *et al.*, 2014; Ikeue *et al.*, 2015; Bawin *et al.*, 2023), which aligns with earlier reports that identified these enzymes in infection sites (Nagar *et al.*, 1984; Johnsen *et al.*, 2015). Pectin, which forms the connection layer between cells and influences the porosity and thickness of cell walls, is particularly targeted for degradation during parasitic infections (Fig. 4). Another cell-wall constituent that appears to be closely regulated in *Cuscuta* infection sites is the hemicellulose xyloglucan (XG). The dual function enzyme xyloglucan endotransglucosylase/hydrolase (XTH) can either modify and strengthen XG linkages with their endotransglucosylation activity or weaken XG-rich cell walls by their hydrolase activity. Both the swelling in the upper haustoria and the intrusive growth of the hyphae require flexible, extensible cell walls, which would fit with XTH’s hydrolytic activity, but it is also conceivable that the surface of mature haustoria are in need of fortification, which would be governed, among others, by XTH’s transglucosylation activity (see Olsen *et al.*, 2016*a*). Since it is difficult to demonstrate the hydrolytic activity *in situ*, this is, however, purely speculative.

Beyond spatial and temporal aspects of expression, cell-wall-degrading enzymes in *Cuscuta* may exhibit substrate promiscuity, as exemplified by endoglucanases that were found to target both cellulose and hemicellulose compounds (Edema *et al.*, 2024). The parallel upregulation of genes coding for BBE-like proteins in more advanced haustoriogenesis stages (Bawin *et al.*, 2023) supports the attractive notion that *Cuscuta* takes precautions to prevent damage-induced defence responses, facilitating a concealed invasion. An important aspect to be considered in this respect is the possible influence of the cellular environment provided by the host in which the haustorium grows, and how it differs from the haustorial chemistry. The possible modulation of the hydrolytic activity of cell-wall-related enzymes, be it via ion or sugar transporters, or additional enzymes, with changes in ion distribution and local pH modifications, is suggested by recent studies and awaits verification. For instance, the apparent tight regulation of calcium at the border between host and haustorial tissues (Förste *et al.*, 2020), and the observation in non-parasitic plants that pH-responsive genes are linked to intracellular calcium (Lager *et al.*, 2010), lend support to a hypothetical scenario where multiple layers of control orchestrate haustorial progression. Much remains to be done to elucidate how the enzymatic arsenal in *Cuscuta* interacts with developmental and immunity components to drive successful haustorial growth and host invasion.

## NUTRIENT FLOW THROUGH THE HAUSTORIUM – A ROADMAP FULL OF GAPS

The main function of haustoria is to enable the uptake of water, inorganic nutrients and, in the case of holoparasites such as *Cuscuta*, also organic molecules from the hosts. This is achieved by establishing a continuity between the vascular system of the host and that of the parasite. It is uncontested that the vascular continuity is achieved by hyphae emerging from the tips and flanks of the haustoria that build a bridge between the host cells on one side and cells in the haustorium that differentiate into vascular cells on the other side (Hartenstein *et al.*, 2023). When still searching for a cell to connect to, these hyphae are undifferentiated, but they differentiate into a matching cell type as soon as they establish contact with the corresponding vascular host cells (Vaughn, 2006). Hyphae intercepting xylem vessels of the host differentiate into xylem-like (xylic) hyphae (Christensen *et al.*, 2003) while those contacting host sieve elements turn into phloic hyphae (Dörr, 1972). Each haustorium can produce both types of hyphae.

Overall, the interactions at the host–parasite interface are highly complex and to date no complete and convincing model exists that describes satisfactorily how the haustorium form–function relationship is regulated, at what timepoint open connections to the host are established, how long they are maintained, which type of hyphae contributes when, and how selective they are. What complicates matters further is that each individual *Cuscuta* plant connects to one or several hosts with tens or hundreds of haustoria. Below, some of the most intriguing mysteries that remain unsolved despite intense research and large progress are debated alongside a summary of the currently available knowledge on the vascular host–parasite connections and the flux of nutrients and other molecules.

### Transport via the xylem

In autotrophic plants, two things are paramount for the uptake and transport of water and inorganic nutrients. (1) A selective barrier formed by the root endodermis with its Casparian strip that forces all solutes through the symplast before reaching the xylem. This gives substrate-specific membrane transporters at both the intake and the export side the possibility to control which minerals are taken up. (2) The possession of leaves with stomata that drive long-distance transport by way of evaporation of water. *Cuscuta* lacks roots and obtains its nutrients exclusively through multiple haustoria alongside its branching shoots that connect directly to the vasculature of the host. Moreover, it lacks stomata-bearing leaves and exhibits an extreme paucity, bordering on absence, of stomata that function in gas exchange on their stems (discussed by Hartenstein *et al.*, 2023) so that the main intake control (i.e. the Caspary strip) and the main known driver for xylem flow (i.e. transpiration) are absent. Nevertheless, there are tracheids that transport the water and minerals that were sequestered from the host vasculature with xylic hyphae. The xylem connection between host and parasite is widely regarded as an open pathway, securing water and mineral supply for the parasites, and unspoken assumptions were made that no selection barriers exist. However, the early analysis of the mineral composition of *Cuscuta* species and their hosts revealed some striking differences in the concentration of some minerals, with the concentrations of calcium (Ca), magnesium (Mg), manganese (Mn), sodium (Na) and boron (B) being much lower in the parasites than in the hosts (Wallace *et al.*, 1978; Saric *et al.*, 1991). In a more detailed analysis of the tissue-specific mineral concentrations in the *C. reflexa–Pelargonium zonale* system using X-ray fluorescence spectrometry (XRF), these early data were confirmed and extended (Förste *et al.*, 2020). While some of the elements (Ca, Mn, bromine, strontium) have very low concentrations in all tissues of the parasite, chlorine (Cl) is found at high concentrations in the haustoria, comparable to those in the host, while the concentration in the stems of the parasite is very low. These findings indicate that mineral transport at the *Cuscuta*–host border is significantly more complex than previously acknowledged. Interestingly, in hemiparasites, which have connections only with the host xylem, but not with its phloem, the mineral composition is much more consistent with that of the hosts (Rozema *et al.*, 1986). A biophysical explanation for the selective exclusion analogous to the function of the Caspary strip does not seem to exist in *Cuscuta*, leaving biochemical reasons to be explored. Unfortunately, a deduction of active transport from the presence or absence of the expression of substrate-specific membrane transporters is not reliable since most transporters are part of large families with overlapping specificities. Therefore, the most parsimonious explanation for the uptake selectivity is that the transport of minerals is accomplished by the phloem or other naturally symplastic (e.g. parenchymatic) connections. Most of the minerals that are depleted in *Cuscuta* (e.g. Ca, Mn, B) are barely mobile or even immobile in the phloem (Etienne *et al.*, 2018), which would be consistent with their low concentration in the parasites if their uptake were via this route. The main exception is Cl, which in the form of chloride is highly mobile in the phloem which would enable its transport into the haustorium. Here the drop in concentrations of this element in the stem tissue indicates a tentative active retention by export from the phloem into the surrounding tissue.

If this scenario were true, and xylem connections may not be active in uptake processes, they must fulfil a different role. Principally, this could also be the opposite of uptake. It has long been a mystery how *Cuscuta* can maintain water homeostasis. Its rapid growth might demand some water, but it is unlikely that this alone can suffice to balance the high intake. An export of excess water that cannot evaporate via stomata in *Cuscuta* is therefore probably an overlooked necessity for the parasite. A similar phenomenon is well known from fruits (Zhang and Keller, 2017), which, exactly like *Cuscuta*, have very low transpiration rates and are very strong sinks for organic material but also need to import minerals. In young, growing fruits both phloem and xylem contribute to the supply of water and minerals while in later stages of fruit development the contribution of the xylem gradually becomes negligible (Choat *et al.*, 2009), which is accompanied by a sharp drop in Ca uptake (Hocking *et al.*, 2016). In some plants the direction of xylem flow can even be reversed during fruit ripening (Keller *et al.*, 2015). The fruit water budget is thus the result of water exchange via the vascular system of the plants and the fruits (Hou *et al.*, 2021). In contrast to the extensive knowledge about water transport in fruits (see the excellent review by Hou *et al.*, 2021) nothing is known about the dynamics of water transport during the life cycle of *Cuscuta*. A thorough investigation of nutrient and water flow dynamics is therefore an imperative task that would have implications for basic scientific understanding of the parasitic strategy as well as for applied aspects involving the transport of molecules for investigative or therapeutic applications [e.g. host-induced gene silencing (Alakonya *et al.*, 2012), virus transmission to hosts (Bhat and Rao, 2020)] and grafting (see section below). To our knowledge, no measurements of the total water potentials nor the osmotic potentials or turgor have been reported for any *Cuscuta*–host interaction. It is also unknown if the direction of water transport can vary between different haustoria or whether it changes during the course of maturation of each individual haustorium. The insufficiently researched contribution of stomatal transpiration to water balance in *Cuscuta* would possibly also need to be revisited. Last, but not least, it would be interesting to test if infection by holoparasitic plants changes the water status of the host plants.

### Transport via the phloem

In contrast to the apoplastic transport in the xylem, phloem transport is based on symplastic movements and mediates the long-distance transport of small organic molecules and larger macromolecules. Symplastic transport necessitates cytosolic connections across cell walls that are realized by plasmodesmata (PD) (Ehlers and Kollmann, 2001; Schreiber *et al.*, 2024). A special case of PD are interspecific secondary PD (iPD) that are formed across already existing cell walls to connect the symplasts of different individuals from the same or different species (Fischer *et al.*, 2021). iPD were identified in a number of parasite–host pairs, mainly in the genus *Cuscuta* and in Orobanchaceae, corroborating the large body of physiological and molecular evidence for symplastic transport between holoparasitic plants and their hosts, but for many parasite–host systems ultrastructural evidence of iPD is still missing (reviewed by Fischer *et al.*, 2021). Although the transport of sugars, amino acids and other small organic molecules over short distances at the host–parasite interface can in theory also be mediated by apoplastic transport, this pathway is generally regarded as too inefficient and slow to account for the scale of uptake that were reported (e.g. Hibberd and Jeschke, 2001), which again is generally interpreted as evidence for phloem connections. Moreover, larger molecules or even viruses that are able to move between the host and the parasite (Hosford, 1967) can only take the symplastic pathway between cells.

In the last three decades, the evidence for an extensive exchange of macromolecules between parasites and hosts has become overwhelming (reviewed by Shen *et al.*, 2023). Proteins, plant hormones, mRNA, micro (mi) and small interfering (si) RNAs, and probably even DNA fragments were shown to be shuttled bidirectionally between both plant types. The first evidence for protein transport from a host (*Nicotiana tabacum*) to a parasitic plant (*Cuscuta reflexa*) was provided by Haupt *et al.* (2001). Here, a green fluorescent protein (GFP) was produced specifically in the phloem of the host plant and shown to be transported into the parasite. Later, GFP movement was also shown for the tomato–*Phelipanche aegyptiaca* system (Aly *et al.*, 2011). However, the enormous scale of protein transport was only realized by proteomics approaches. The extent of transport, with hundreds to more than 1000 proteins that were found to move between *Cuscuta australis* and *Arabidopsis* or soybean hosts (Liu *et al.*, 2020), was another surprise, next to the apparent lack of directional preference: a large number of host proteins were detected in *Cuscuta* and large quantities of *Cuscuta* proteins were found in the host plants. Although the transported proteins do not seem to be degraded immediately and may therefore be stable, a clear physiological role in the interaction partner has in most or even all cases not been demonstrated. However, a significant change in the composition of the transported proteins under nutrient stress might indicate a role of these proteins in stress response and signalling (Zhang *et al.*, 2024).

DNA transfer has, to our knowledge, not been directly observed between parasites and hosts. However, movement of DNA molecules can be one of the possible hypotheses for the frequent occurrence of HGT between host plants and parasitic plants (Davis and Xi, 2015). Analyses of the transcriptomes and complete nuclear genomes of several parasitic plants revealed the presence of up to several hundred HGT candidates in different species (Vogel *et al.*, 2018; Yang *et al.*, 2019; Yoshida *et al.*, 2019; Cai *et al.*, 2021).

Over the last two decades it has, moreover, become apparent that neighbouring and distant plant cells exchange RNA via the long-distance phloem-based route (Kehr and Kragler, 2018). All major classes of RNA molecules (i.e. small and large non-coding RNAs, mRNAs, tRNAs and rRNAs) are transported. For a few of the mobile small non-coding RNAs and mRNAs, a role in coordination of plant development, stress response and other processes has been determined (Kehr and Kragler, 2018). A transfer of mRNAs from hosts to a parasitic plant was first shown by Roney *et al.* (2007) for *C. pentagona* growing on *Cucurbita maxima* or *Solanum lycopersicum*, but other studies soon revealed the bidirectionality of their transport, and have demonstrated the huge scale at which this happens with several hundred to several thousand transcripts being transported between parasites and hosts (Kim *et al.*, 2014; Li *et al.*, 2022). However, how many of these mobile RNAs are *de facto* translated into proteins in the recipient plants has yet to be determined (Park *et al.*, 2021).

In contrast, functional confirmation in the context of gene expression control for shuttled small non-coding RNAs has been achieved in various instances (Tomilov *et al.*, 2008; Shahid *et al.*, 2018; Johnson and Axtell, 2019; Shen *et al.*, 2023). These studies indicate that manipulations of the host transcriptome by *C. campestris* are likely to facilitate successful infection and that miRNAs are employed as virulence factors during parasitism. *Cuscuta campestris* has been shown to express various miRNAs during *Arabidopsis* infection, where the majority of these were 22 nucleotides in length and targeted many *Arabidopsis thaliana* mRNAs via further processing and siRNA-mediated cleavage (Shahid *et al.*, 2018). Zhou *et al.* (2021) analysed differentially expressed genes and miRNAs and found that 23 pairs of differentially expressed miRNA–mRNA pairs were associated with hormone signal transduction, ribosome and plant–pathogen interaction pathways during *Cuscuta australis* infection. Not only does RNA interference (RNAi) act at the host–parasite interface but it was also shown that some small RNAs move long distances into the host plant (Subhankar *et al.*, 2021). The exact reason and effects this has on host plants is still unclear and needs to be investigated further but it may prime the host plant for secondary infection by *Cuscuta*. Interestingly, *C. campestris* contains many novel miRNAs, including a new miRNA that may have been derived from an HGT event, and it seems to have retained miRNAs that have been lost in other Solanales, while some more conserved miRNAs have become obsolete (Zangishei *et al.*, 2022).

Strangely, although transport of various biomolecules has been confirmed to occur both into and out of *Cuscuta*, the mechanistics of it has not been revealed. The flow of the phloem stream in the host plant always follows the sink strength. With the general assumption that *Cuscuta* is a strong sink, the macromolecules transported in the phloem would be expected to flow only in the direction of the parasite, leaving it a mystery how export back to the host is accomplished. The complex exchange patterns could be theoretically explained by modulations in sink strength in regions of the parasite, which could be spatially or temporally separated from the areas responsible for nutrient intake. More research is needed to trace flow patterns in *Cuscuta* and also investigate the underlying regulation of these processes.

## THE HAUSTORIUM AS A NATURAL GRAFT

Interspecific vascular connections are formed not only between parasitic plants and their hosts, but also between scion and host during plant grafting. In fact, the haustorium has been compared to a ‘perfect graft’ (Kuijt, 1969) because in mature infection sites, the tissue of parasite and host blend perfectly into each other. The fact that parasitic plants like *Cuscuta* are further able to connect with a wide variety of host plants while grafting is successful with only a very limited range of plant species renders the processes that unfold during haustorial infection into a potential treasure trove for genes that could widen grafting compatibilities. In reverse conclusion, attempts to develop effective strategies for mitigating the impact of parasitic plant infestations on agricultural productivity could take inspiration in the molecular obstacles that hinder the re-joining of the vascular tissue in incompatible grafts. The regulatory roles of phytohormones like auxin, cytokinin and ethylene, and their influence on vascular development have been highlighted in both grafting and parasitic plant interactions. Also, notably, both processes involve the differential expression of genes associated with the manipulation of cell walls and with cell–cell adhesion. This last section will therefore venture into the field of grafting and compare the processes underlying vascular reconnection in the establishment of both interfaces, shedding light on their molecular intricacies and examining apparent parallels between the haustorial infection and grafting processes.

### Xylem-specific factors

**Fig. 5.**
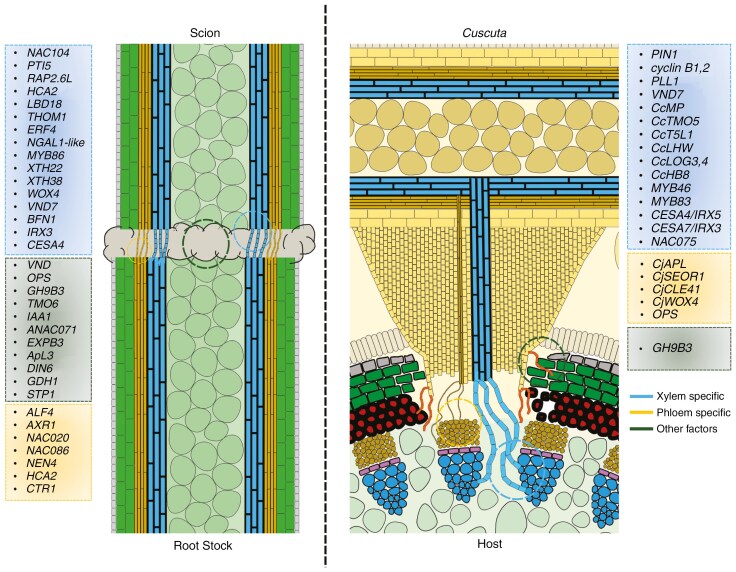
Comparison of genes activated during grafting (left) and haustorium formation (right). Blue colour represents newly formed xylem strands between scion and rootstock (grafting) and between parasite and host (parasitism). Genes associated with their formation are listed in the blue boxes. Orange-brown strands represent phloem-like vessels which have formed during grafting and parasitism, respectively. Genes differentially expressed and associated with phloem formation and reconnection during grafting and parasitism, respectively, are shown in the orange box. The dark-green box contains genes differentially expressed during grafting or parasitism that are not directly related to phloem or xylem differentiation.

*Cuscuta* is able to induce searching hyphae *in vitro*, but the differentiation of xylem-like tissue that forms a xylem bridge was reported to happen only upon encountering the host xylem (Kaga *et al.*, 2020). The potential for xylem differentiation, however, was already evident before penetration, as specific genes were upregulated at 57 h after infection (hai), including several transcription factors of the bHLH, HD-ZIPIII and ARF groups, as well as genes involved in cytokinin synthesis and perception/signal transduction. Additionally, some genes were upregulated to inhibit vascular stem cell proliferation due to negative cytokinin signalling at 57 hai (Kaga *et al.*, 2020). The VASCULAR-RELATED NAC-DOMAIN7 (*VND7*) was upregulated at 87 hai, as was an orthologue of a tentative *VND7* regulator, *NAC075* (Endo *et al.*, 2015; Kaga *et al.*, 2020). Genes downstream of *VND7*, such as *CELLULOSE SYNTHASE A4/IRREGULAR XYLEM 5* (*CESA4/IRX5*), and *CESA7/IRX3*, which in *Arabidopsis* have been shown to be involved in the formation of secondary cell walls (Taylor *et al*., 2000, 2003) and their regulators, *MYB46* and *MYB83* (Zhong *et al.*, 2007*a*; McCarthy *et al.*, 2009), showed similar expression profiles (Kubo *et al.*, 2005; Kaga *et al.*, 2020). Furthermore, Kaga *et al.* (2020) identified differentially expressed genes for lignin biosynthesis, as well as cysteine and serine peptidase genes, suggesting that *C. campestris* prepares for xylem differentiation in haustoriogenesis, driven ostensibly by well-known factors (Fig. 5). It is not known if the differentiation of xylic hyphae and other xylem bridge cells in this parasite precedes establishment of the phloem connection or if it is the other way round.

In contrast, xylem reconnection between the rootstock and scion during graft formation typically occurs after phloem reconnection, normally around 7 d after grafting (DAG) (Melnyk *et al.*, 2015). Thomas *et al.* (2021) identified several potential hub genes involved in plant vasculature reconnection, particularly for xylem reconnection, during the grafting of tomato and pepper (Fig. 5). However, it shouldbe noted that these hubs differed to some extent depending on the choice of grafting partners. However, through the use of correlation networks, it was shown that most of them converged to regulate two *XTH* genes, *XTH22* and *XTH38* (Thomas *et al.*, 2021), homologues of which play a role in *Cuscuta* haustoriogenesis (Olsen *et al.*, 2016*b*) (see also earlier section in this review).

In pepper–pepper homo-grafts, moreover, *LBD25* was found to be upregulated at 5 DAG. As mentioned earlier, this transcription factor was also identified in *Cuscuta* as a potential regulator of the development of searching hyphae (Jhu *et al.*, 2021*b*) which makes this gene a very interesting candidate in the context of vasculature reconnections. Incompatible pepper–tomato (and vice versa) hetero-grafts showed perturbations of gene networks in comparison to those seen in successful homo-grafts, like that of pepper on pepper (Melnyk *et al.*, 2018; Thomas *et al.*, 2021). One of the genes that differed in these interactions was the tomato homologue of *WOX4*. This gene regulates VNDs and NAC transcription factors (NSTs), both of which play a role in xylem differentiation (Kubo *et al.*, 2005; Zhong *et al.*, 2007*b*; Kondo *et al.*, 2015; Turco *et al.*, 2019; Thomas *et al.*, 2021). In brief, it seems that when cells prepare for cell re-differentiation, and specifically for xylem formation, the expression of the *WOX4* gene is needed in at least the scion or rootstock tissue for successful xylem formation and reconnection, and its absence causes graft incompatibility. *WOX4* function is another feature that grafts and haustorial infections have in common as this gene was found to be expressed in *Cuscuta japonica* haustoria (Shimizu *et al.*, 2018), showcasing that there are multiple overlaps in key regulators during the formation of interspecific cellular connections, and that even more commonalities may be uncovered with more research.

### Phloem-specific factors

Unfortunately, molecular studies shedding light on the phloem connections between *Cuscuta* and their hosts are scarce. In *C. japonica*, two marker genes for phloem development, *ALTERED PHLOEM DEVELOPMENT* (*CjAPL*) and *SIEVE ELEMENT OCCLUSION-RELATED 1* (*CjSEOR1*), are expressed in intruding hyphae, suggesting symplastic connectivity of phloem conductive cells, with evidence of host-derived substances translocating via distinct conductive cells (Bonke *et al.*, 2003; Knoblauch *et al.*, 2014; Shimizu *et al.*, 2018). *In situ* hybridization for *CjCLE41* (phloem-specific differentiation), meanwhile, overlaps with *CjWOX4* (xylem-specific differentiation), implying a potential delay in the development of haustorium-derived phloem compared to conventional vascular development (Hirakawa *et al.*, 2008; Shimizu *et al.*, 2018).

The process of phloem reconnection during grafting has definitely emerged as a crucial step in ensuring the viability of the graft junction. Research indicates that this reconnection occurs before xylem reconnection, typically around 3 DAG, orchestrated by the action of genes like *ALF4* and *AXR1*, which play pivotal roles in auxin sensing and are particularly crucial for phloem reconnection specifically below the graft junction (in the rootstock), rather than in the scion (Melnyk *et al.*, 2015). The expression of phloem-associated markers peaks at 72 h after grafting, aligning with the expected timing of phloem reconnection (Yin *et al.*, 2012; Melnyk *et al.*, 2015, 2018). Sequential activation of phloem markers such as *NAC020*, *NAC086* and *NEN4* (representing early-, mid- and late-phloem development, respectively) mirrors the phloem transcriptional activation cascade observed in developing roots and leaf vasculature (Furuta *et al.*, 2014; Kondo *et al.*, 2016; Melnyk *et al.*, 2018). Enhanced *HCA2* activity (as seen in *hca2* mutants) in the rootstocks of grafts has been shown to improve phloem reconnection rates, whereas suppressing *HCA2* targets has the opposite effect (Guo *et al.*, 2009; Melnyk *et al.*, 2018). While cytokinin and ethylene signalling have been implicated in vasculature development, their roles in phloem reconnection during grafting appear to be comparatively minor, with auxin signalling taking centre stage (Melnyk *et al.*, 2015).

### Other factors

Other factors beyond the gene networks involved in vascular reconnection can significantly influence the success of grafting. Comparative transcriptomic studies of graft junctions between tobacco–*Arabidopsis* and *Glycine max*–*Arabidopsis* highlighted the significance of β-1,4-glucanase of the glycosyl hydrolase 9B (*GH9B*) family in interfamily graft success (Notaguchi *et al.*, 2020). β-1,4-Glucanases exhibit cellulolytic activities and play roles in cellulose digestion, cell-wall relaxation and cell-wall construction throughout plant growth processes (Cosgrove, 2005; Lewis *et al.*, 2013). Knockdown via VIGS (virus-induced gene silencing) targeting and knockout via CRISPR (clustered regularly interspaced short palindromic repeats) of *NbGH9B3* led to a significant decrease in interfamily grafting success in tobacco, highlighting its necessity in such grafts (Notaguchi *et al.*, 2020). Further investigation into the expression of *GH9B3* homologues in homo-grafts of other plant species revealed expression at 1 DAG in all dicot plants (Roodt *et al.*, 2019; Notaguchi *et al.*, 2020). The only monocot in the tested set that is known to lack cambial activity in the stem lacked expression of this gene. Additionally, overexpression of tobacco *GH9B3* in *Arabidopsis* significantly enhanced grafting success (Notaguchi *et al.*, 2020). In *Cuscuta*–host interactions, *GH9* genes of the B-type have recently been shown to be induced during haustoriogenesis, with the fold-change, by which they were upregulated, depending on whether their interaction partner was a susceptible or semi-resistant live plant or a mock host (Edema *et al.*, 2024). These findings underscore the importance of *GH9B*-type genes in the establishment of an interspecies interface, be it a graft junction or an infection site.

Genes serving as markers for cell division were activated in the scions as early as 12 h, and by 24 h, they were also activated in the rootstock (Melnyk *et al.*, 2018). Control genes (such as housekeeping genes) displayed no differential expression patterns in either the scion or rootstocks (Czechowski *et al.*, 2005; Melnyk *et al.*, 2018). A multitude of genes involved in various processes such as vasculature development and cell division exhibited asymmetric expression, predominantly in the scion region, which appears to be a recurring theme in tissue reconnection and graft junction formation (Asahina *et al.*, 2011; Melnyk *et al.*, 2015, 2018). Further transcriptional analysis suggests that asymmetric gene expression occurs during the early post-grafting stages and gradually becomes more symmetric as the graft junction forms and heals, culminating in stable vascular reconnection, resumed transport of hormones, sugars and proteins, and complete healing of the graft site (Melnyk *et al.*, 2018). Whether asymmetric gene expression happens during host infection by *Cuscuta* has to our knowledge not yet been assessed. However, the infection differs from the grafting process in that the perturbation resembles a detour and not a complete block of metabolite traffic. The accumulation of substances along the graft junction from both sides that exists until homeostasis is achieved through vascular reconnection is therefore absent in the infection process. As mentioned in the previous section, a detailed map of the flow patterns in infected hosts as well as in the parasite may shed more light on the intricate nature of the interplay between nutrient, metabolite and gene expression gradients.

### Insight from other parasitic plant models

While all parasitic plants have evolved xylem connections to the host, the transition of searching hyphae to phloem-conducting elements varies strongly across species. Although both *Cuscuta* and *Phtheirospermum* exhibit distinct procambium-like cell development preceding xylem and phloem differentiation, *Phtheirospermum* seems to lack phloem conducting elements (Spallek *et al.*, 2017; Wakatake *et al.*, 2018). Similarly, *Phelipanche aegyptiaca* (formerly known as *Orobanche aegyptiaca*) seems to develop immature phloem conducting elements in the haustorium (Ekawa and Aoki, 2017), while mature phloem conducting tissue, containing mature sieve elements, is found in the haustoria of *Orobanche crenata* and *O. cumana* (Dörr and Kollmann, 1995; Krupp *et al.*, 2019).

Observations at the interface between *Phtheirospermum japonicum* and *Arabidopsis thaliana* revealed thinned cell walls, suggesting that digestion occurred at the interface, similar to the graft boundary between *Arabidopsis* and *Nicotiana* (Kurotani *et al.*, 2020). *Phtheirospermum japonicum* demonstrated high success rates in grafting to various species as rootstock and scion, except for two Fabaceae species (Kurotani *et al.*, 2020). Principal component analysis (PCA) of RNA-sequencing (RNA-seq) data from *P. japonicum*–*Arabidopsis* parasitism and grafting showed significant differences (Kurotani *et al.*, 2020). Genes related to xylem differentiation and phloem differentiation were upregulated in both grafting and parasitism, while other genes were upregulated only during grafting (Kurotani *et al.*, 2020). Further RNA-seq comparison and RNAi experiments revealed that knockdown of the glycosyl hydrolase *PjGH9B3* in *P. japonicum* significantly reduced the successful xylem connection ability of *P. japonicum* to a host plant, similar to the findings from grafting outcomes (Kurotani *et al.*, 2020; Notaguchi *et al.*, 2020). Parasitic plants exhibit a greater ability to manipulate plant cell walls to gain access and differentiate to attach to the host plant vasculature. This was evident as the non-parasitic *Lindenbergia philippensis* failed in grafting, while parasitic equivalents showed great success (Kurotani *et al.*, 2020). Thus, similarities exist between grafting connection and parasitic plant connection in terms of cell–cell adhesion.

## CONCLUSIONS AND FUTURE DIRECTIONS

Although much progress has been made in understanding the process of haustoriogenesis, this has been biased towards molecular processes, informed by transcriptomics, and microscopical descriptions. The intricate processes involved in parasitic plant infection and establishment of vascular connections with host plants that have to date been revealed have highlighted the remarkable adaptability and evolutionary strategies employed by these organisms. The mechanistic understanding of the haustorial infection process, and with it also the establishment of interspecific cytoplasmic continuity, has lagged behind, leaving a number of unanswered questions. Unveiling how *Cuscuta* is able to maintain a strong sink force despite no apparent possibility to dispose of the large quantities of water that would theoretically accompany the flow of nutrients is one of the topics that needs to be addressed. For this, attention needs to shift from a focus on individual haustoria to the entire haustorial system of a plant. Some recently published studies of this type (e.g. Zhang *et al.*, 2024) help to set the stage for a more holistic understanding of the parasitic concept, but many further approaches will be needed.

The exploration of haustorial formation in parasitic plants, particularly *Cuscuta*, reveals a sophisticated process of interspecific vascular connection that parallels and often surpasses traditional grafting. The haustorium’s ability to seamlessly integrate with various host plants highlights potential genetic and molecular pathways that could broaden grafting compatibilities across plant species. Future research should focus on unravelling the molecular mechanisms underlying the ability of *Cuscuta* to manipulate host vascular systems, with particular emphasis on the roles of specific transcription factors, phytohormones and cell-wall-related enzymes. Comparative studies between successful parasitic plant infections and grafting in diverse plant species could identify key genes that enhance graft compatibility and stability. Ultimately, these findings could lead to novel strategies for improving agricultural practices, including enhanced grafting techniques and more effective management of parasitic plant infestations.

With the trend in transgenic technology and the progress with obtaining viable transformed *Cuscuta campestris* (Adhikari *et al.*, 2024), progress in some or all of the areas mentioned throughout this review can be expected to become faster and more targeted.
